# New Public Management and hospital efficiency: the case of Norwegian public hospital trusts

**DOI:** 10.1186/s12913-023-10479-7

**Published:** 2024-01-05

**Authors:** Nils Arne Lindaas, Kjartan Sarheim Anthun, Jon Magnussen

**Affiliations:** 1 Department of Public Health and Nursing, Norwegian University of Science and Technology, 7491 Trondheim, P.O. Box 8905, Norway; 2 Department of Health Research, SINTEF Digital, 7465 Torgaarden, Trondheim, P.O. Box 4760, Norway

**Keywords:** Hospital, Efficiency, New Public Management, Healthcare, Reform

## Abstract

New Public Management-inspired reforms in the Norwegian hospital sector have introduced several features from the private sector into a predominantly public healthcare system. Since the late 1990s, several reforms have been carried out with the intention of improving the utilization of resources. There is, however, limited knowledge about the long-term, and sector-wide effects of these reforms. In this study, using a panel data set of all public hospital trusts spanning nine years, we provide an analysis of the efficiency of hospital trusts using data envelopment analysis (DEA), as well as a Malmquist productivity index. Thereafter we use the efficiency scores as the dependent variable in a second-stage panel data regression analysis. We show that during the period between 2011 and 2019, on average, efficiency has increased over time. Further, in the second-stage analysis, we show that New Public Management features related to incentivization are associated with the level of hospital efficiency. We find no association between degree of competition and efficiency.

**Supplementary Information:**

The online version contains supplementary material available at 10.1186/s12913-023-10479-7.

## Introduction

New Public Management (NPM) inspired reforms have been prominent in public institutions in European and other OECD countries in the last 30 years. NPM has stressed the importance of efficient utilization of scarce public resources, by adopting management practices from the private sector, partly with the assumption that public organizations will not be efficiently run without incentives [1, 2].

For the past 20 years hospitals in Norway have been organized as state owned trusts. There is a two-level system where four state owned regional health authorities (RHA) are responsible for the provision of hospital services in the region. Services are mainly provided in RHA-owned hospital health trust, but also to some extent contracted out to private providers. There is free choice of hospital, and a mixed system of per capita and activity-based financing. This provides an environment of regulated competition with an arms-length form of governance. The role of the state primarily is through legislation, annual budgets, and steering documents. The model is criticized by some for lack of transparency and democratic control and for putting too much emphasis on economic issues rather than quality and equity.

The purpose of this paper is to examine the association between two specific features of this model and the performance of hospitals. First, in the Norwegian model, hospitals bear the full financial responsibility of investments. Thus, the cost of both buildings and equipment must be borne in full by the hospital. The idea is that this will provide an optimal mix of capital and labor and thus a more efficient utilization of resources. Also, it provides a strong incentive for hospitals to improve performance in order to raise funds to cover the cost of future investments. Second, the competitive environment that follows from the combination of free choice of hospital and activity-based financing, further provide incentives for improving performance.

In our analysis, we utilize a panel data set from 2011 to 2019, a period of nine years. We perform a two-stage analysis, where we first use data envelopment analysis (DEA) to establish hospital-specific measures of efficiency. The efficiency scores from the DEA are then used as dependent variables in a second-stage regression analysis. We are not looking at causal effects of reforms, but rather investigating associations between NPM-related variables and hospital efficiency in a relatively stable period where reforms have had long time to settle.

The paper is structured as follows. First, we elaborate on NPM and the related reforms in the Norwegian hospital sector. This is followed by a brief review of past studies. Next, we describe our empirical strategy for the second-stage analysis and the dataset, variables and methods applied. The results are then presented and discussed before we make some concluding remarks.

### New Public Management in the Norwegian hospital sector

NPM-inspired reforms usually introduce new organizational, structural, or managerial tools or changes into public organizations. Even though there is a lack of a precise definition of NPM, several scholars have argued that policy measures can be grouped under the three main themes disaggregation, incentivization, and competition [2–4]. In Table 1 we summarize the main recent policy reforms in Norway and connect them to these main themes.

**Table 1:** Reforms in the Norwegian hospital sector and their associated NPM-features

Reform and year	Description	NPM feature
Hospital ownership reform (2002)	Hospitals organized as independent health trusts with control over both capital and personnel.	Disaggregation, incentivization
Activity-based financing reform (1997)	Introduction of a prospective payment system based on diagnosis-related groups (DRG).	Incentivization
Patient choice reform (2001) Free treatment choice reform (2015)	Patients can choose where to receive treatment. The choice includes both public hospital trusts and private hospitals.	Competition

*Disaggregation* within NPM usually refers to the separation or decoupling of the purchaser and provider of services [2]. In healthcare, the purchaser is often a public body (state, regions, or counties) that finances the service, while the providers are the hospitals (private or public) that deliver the service. This is expected to make previously bureaucratic organizations “more flexible, controllable and manageable by professional managers” [5, p. 335]. It is further assumed that it will strengthen accountability with clearer lines of control [6]. In practice, this disaggregation usually happens through a reshaping of organizations into agencies, and therefore increased agencification is often a result of disaggregation [2]. The hospital ownership reform in Norway in 2002 saw the ownership of hospitals transferred from 19 counties to the state, while at the same time disaggregating the governance of hospitals to five (eventually four) regional health authorities (RHAs) organized as trusts. RHAs own the hospitals, also organized as trusts. Whereas investments were financed separately pre-reform, hospitals were now made responsible for the full financing of both capital and personnel [7, 8]. The RHAs fill a double function as both purchaser and provider of hospital services [9]. The balance between central state ownership control and decentralized autonomy after the reform has been discussed by Lægreid et al. [10].

*Incentivization* is assumed to work as motivation for both cost saving and improved efficiency by rewarding certain operations, activities, and performances [2, 3]. Such rewards can be both in the form of rewarding specific activities over others, or through more general incentive programs aimed at increasing overall efficiency. In the case of Norway, two types of incentivization can be highlighted. First, the introduction of private sector accounting principles into the public health trusts, and the health trust bearing the full cost of investments provides a strong incentive for a better and more holistic use of the resources. Second, a financing model where the global budgeting of hospitals was replaced by a system of partly activity-based financing (ABF) based on the diagnosis-related groups (DRG) system. This introduced a hybrid financing system where the individual health trusts are financed by the RHA by a combination of ABF and global budgets. The global budgets are based on characteristics of the catchment area of the health trusts related to demographic, health, and social factors [11]. The reform aimed to provide hospitals with incentives to increase activity as well as to improve efficiency [12]. When first implemented, 30% of the budget was ABF and 70% was global budgets. In the period 2011–2019 analyzed in this paper the share of ABF has varied between 40 and 50%.

*Competition* implies introducing choice, internal markets or contracting the provision of services out to private providers [2]. This will, according to the theory, “inject competitive pressures into the public sector” [6, p. 286], and thus lead to better performance. Measures to increase competition were introduced into the Norwegian hospital sector through two patient choice reforms in 2001 and 2015. The patient choice reform in 2001 allowed patients to choose hospital for elective treatments [9]. The choice was limited to public hospitals with the same degree of specialization and private providers who were under contract with the RHA. In 2015, the free treatment choice reform, further expanded the patient’s scope of choice by also including all approved private providers regardless of whether they had contracts with the RHAs [13]. Even though there is competition “on paper”, there are some practical problems with competition in the Norwegian setting as argued by Brekke and Straume [9]. These are primarily related to patients’ ability to make informed choices on one side, and Norway’s geographical structure with large distances on the other side.

### Past studies

There is a large literature using data envelopment analysis (DEA) to measure hospital efficiency [14]. Although there is also a vast number of studies looking at the association between various reforms and efficiency, the literature explicitly looking at the association between NPM-inspired reforms and efficiency is scarce.

One study explicitly looking at direct consequences of NPM-reforms is Alonso et al. [5]. Within Madrid, they compared the efficiency of hospitals that had implemented an NPM-inspired reform with hospitals that had not. Their findings showed that there was no difference in efficiency between the hospitals subjected to and those not subjected to reforms. Using similar methods to this present study, Anthun et al. [15] investigated productivity in Norwegian hospitals in the period 1999 to 2014. They found a productivity growth in Norwegian hospitals of 24.6% points for the whole study period. Most of this growth are ascribed to the immediate period after the 2002 hospital ownership reform. From 2003 to 2014 the average observed productivity growth was substantively lower than in the full period, with below 0.5% points annual increase (vs. 1.5% in the full period).

Despite the limited actual competition in the Norwegian hospital sector, Ringard and Hagen [16] showed that even though few patients used the right to choose where to receive treatment, those who did choose waited on average 11 weeks less than those who did not.

Several studies from the English NHS have shown a positive association between hospital competition and measures of hospital quality [17–19]. There are also several studies investigating the effect of competition on *efficiency*, but the findings seem to be dependent on both context and methods applied. Longo et al. [20] found a positive association between competition and several indicators of efficiency, but they also found an increasing number of elective operations to be canceled. Rosko and Mutter [21] found that hospital competition within American counties had a positive association with cost-efficiency. In a two-stage DEA study looking at the association between competition and efficiency, Nedelea and Fannin [22] found that decreased market competition was significantly associated with decreased technical efficiency in American hospitals. Tiemann and Schreyögg [23] found no effect of competition on hospital efficiency in their study of German hospitals after privatization, also measuring competition using HHI with counties as the market area. A more recent study from Germany however, found that hospitals in the least competitive areas tended to be more efficient [24]. This study used HHI with the marked area as a 32 km radius of each hospital.

### Empirical strategy

The focus of our analysis is potential association between the degree of both incentivization and competition, and hospital efficiency. Thus, we need to both provide a measure of efficiency and operationalize these two features for use in the second-stage analysis. We begin by describing the variables used in the second stage analysis.

#### Incentivization

A departing point for our analysis is that the responsibility of hospitals to fund their own investments creates an incentive for efficiency. Hospitals can obtain funding for new investments through (non-payable) depreciation costs and operating surpluses. Additional funding can be obtained through state loans, but these can only cover 70% of the investment costs. Thus, a budget surplus is essential in order to fund investments. Lindaas et al. [25] analyzes variations in size and accuracy of budget surpluses in Norway but does not consider the relationship between surplus budgeting and efficiency.

Hospitals will provide a measure of expected budget surplus in the beginning of the year. By improving efficiency, actual surplus may exceed expected surplus, and vice versa. We have considered two alternative ways of how to include surplus budgeting. The first option is to include a variable measuring the relative size of the budgeted result. This variable will then work as a proxy-variable that measures the health trust’s intention of following the incentives given by the reforms. The second option is to use the deviation between actual result and budgeted result as a measure of how well the health trusts follow their own plans. In this study we have decided to use the former alternative for two reasons. First, since the health trust’s income for the most part is known in advance, a variable measuring budgeted surplus will provide a good measure of how the health trusts responds to the incentivization from the reform. Second, using the latter option will most likely introduce endogeneity problems into the model, since the actual operating costs are used in the estimation of the efficiency score used as dependent variable. Our hypothesis is that higher budgeted results are associated with increased efficiency levels. We measure the budgeted result as a percentage of total operating costs in order to secure comparability across health trusts of different sizes. It is also reasonable to believe that already accumulated savings from previous years can affect the size of the budgeted result when planning larger investments. It would thus be preferable to include some data capturing this, but this is unfortunately not available. As a sensitivity analysis, we test the inclusion of a lagged version of this variable, i.e., whether the planned surplus last year affects the efficiency the following year.

Optimally, we would also include a variable measuring the share of ABF as a measure of incentivization, but due to the limited variation both between hospitals and across years, this is not a feasible alternative.

#### Competition

We capture the degree of competition by using a Herfindahl-Hirschman index (HHI) to measure *market concentration*. There are different approaches used to define the “market” area in which the hospital operates. The most common are through geographical distance and radius, or through (the geopolitical) regions or counties. There is thus a choice between defining the market area from a hospital-specific perspective or an overall market perspective [26]. According to Tiemann and Schreyögg [23], there is little difference in which we use. For the Norwegian case however, we believe that it will matter which one we use. Norway is a country with relatively few inhabitants, combined with a rather large geography. Therefore, in certain areas there would be no feasible way of defining the market area as a geographical distance, as some health trusts would have no other health trusts to compete with. By using the RHA, we are ensured that all health trusts have some other health trust to “compete” with. We thus use the RHAs as the market area. We measured this, for each health trust, as the share of hospital activity in each major diagnostic category (MDC), within the RHA. The final measure is the weighted sum of market share in each MDC.

#### Other control variables

In the first stage of the analysis, we obtain measures of the efficiency of the health trusts. However, we believe that the level of efficiency can depend on environmental variables that capture structural and organizational characteristics of the health trusts. Thus, we believe they are important factors to control for in the analysis. We include the following three control variables: *Personnel mix, hospital structure* and *research output*. While they are not directly related to NPM, all of these variables are still relevant from a policy perspective.

P*ersonnel mix* is measured as the percentage share of full-time equivalents (FTE) in the health trusts working within administration and management, as well as office personnel. Past studies have included different configurations of personnel mix as second stage variables [23, 27, 28]. Since all health trusts have the same tasks, we believe that variation across health trusts within this variable could, to some degree, capture some of the managerial decision-making disaggregated to the health trusts.

An initial effect of the ownership reform was several mergers. The number of hospitals was reduced from 43 in 2002 to 20 in 2011 [29]. Thus, the reform led to a hospital structure where health trusts consist of between 1 and 6 hospitals. In a study of 17 hospital mergers that took place between 1992 and 2000, Kjekshus & Hagen [30] found that most mergers did not have the intended effects on efficiency, while Kjekshus et al. [31] found a significant effect of mergers on long-term sickness among hospital employees. We include a variable that measures *hospital structure* as the activity-adjusted number of hospitals within each health trust. This variable is calculated as the number of hospitals within each health trust divided by the total DRG-points produced by the health trust within the year.

Finally, we include a variable capturing *research output*. This variable is based on the Norwegian system for measuring research output. Each research publication is weighted based on the type of publication and publication outlet. This variable is also made relative to size by dividing the research output points by the total DRG-points produced within the year.

The descriptive statistics for the second stage independent variables are presented in Table 2.

**Table 2:** Descriptive statistics for second stage independent variables

Variable	Obs. (health trust-years)	Mean	Std. dev.	Min	Max
Budgeted result	171	0.805	1.248	-4.915	3.510
Market concentration (HHI)	171	0.301	0.103	0.152	0.493
Personnel mix	171	17.864	1.948	13.589	23.586
Hospital structure (relative to DRG)	171	0.056	0.042	0.010	0.178
Research output (relative to DRG)	171	1.989	2.141	0.034	10.343

## Methods

Using the ideas initially suggested by Farrell [32], data envelopment analysis (DEA) was first presented by Charnes et al. [33]. DEA is a non-parametric method used for computing efficiency and/or productivity scores for decision-making units (DMU), in this case for hospitals. It is a data-driven method used to construct an efficiency frontier based on the ratio between inputs and outputs for the DMUs [34]. Since this is a non-parametric method, the frontier is a measure of the best observed practice among only the included DMUs. The efficient DMUs, which are located on the frontier, will have a score of 1, while the inefficient DMUs will have a score below 1, based on their distance from the frontier. Each DMU is compared to a “peer”, which is a DMU with similar inputs and outputs. An advantage of DEA is the lack of restrictions. However, it is susceptible to measurement errors.

We define four output categories: emergency care inpatient discharges, elective care inpatient discharges, day care treatment, and outpatient visits. Within each output, the case mix is adjusted for by using the DRG-system and corresponding relative weights [15]. Input is measured as total operating costs (in real terms) used for patient treatment, as well as total capital costs. In addition to the four output categories, we could also have used research output as an output category. However, since the input variables only covers operating costs related to patient treatment (together with capital costs), we believe that it is more fitting to add this variable as a control variable in the second stage, rather than as an output variable in the first stage. We assume variable returns to scale and use an input-oriented approach. We also apply bootstrapping, which allows us to estimate confidence intervals and statistical uncertainty.

**Table 3:** Descriptive statistics for DEA analyses

Variable	Obs. (health trust-years)	Mean	St. dev.	Min	Max
Inputs					
Total operating costs (1000 NOK)	171	4,027,007	3,017,814	963,514	15,900,000
Capital costs (1000 NOK)	171	281,818	212,436	52,450	1,089,819
Outputs					
Emergency inpatient discharges	171	33653.95	17792.81	7664.01	79273.42
Elective inpatient discharges	171	19951.85	20878.77	2503.70	102441.1
Day care treatment	171	3875.73	2385.53	679.70	12865.22
Outpatient visits	171	15957.31	11684.06	2340.016	59774.52

Due to the combination of a low number of hospitals (19 in our sample) and the panel structure of the data, we need to choose between pooling the data or running separate DEA-analysis for each year. Some studies have dealt with this by running the DEA on data that is pooled over time [22, 35]. By pooling the data, the sample size can be substantively increased, which will make for more precise statistical estimations. This, however, builds on the assumption that the technology does not change over the study period [35]. The longer the study period is, the less realistic this assumption will be. According to Cavalieri et al. [36], it is therefore more reasonable for studies with longer time-series to use a “contemporaneous frontier approach”, where a yearly DEA frontier is estimated based only on data from that single year. This approach is also used in other studies with longer time-series [23, 37]. The contemporaneous frontier approach might introduce another problem because it is more difficult to identify whether an efficiency change in a single DMU is due to changes in the DMU or changes in the frontier [14].

We use the Malmquist Index (MI) to test for changes in the technology frontier over time, as the MI allows us to measure both efficiency change and technical change [34]. Using MI with DEA to measure total factor productivity change was first proposed by Färe et al. [38]. Anthun et al. [15] found that between 1999 and 2014, there was a positive front shift in half of the years, and a negative front shift in the other half, indicating that there was not a substantial overall shift over this period. We will use the result from the MI as an indication of whether the pooled or the contemporaneous approach are the most suitable approach for our second-stage analysis. While this is not an optimal solution, the second stage regression analysis will still provide reliable estimates of the effect on the observed efficiency scores when controlling for time-effects.

In the second stage, we follow Simar and Wilson [39] and use a bootstrapped truncated regression model, with the bootstrapped DEA estimates from the first stage as the dependent variable. Du et al. [40] uses both the pooled and the contemporaneous frontier approach together with a Simar and Wilson second stage-analysis of panel data in their study. They find, both in Monte-Carlo simulations and with empirical data, that if the difference between the frontier between years is small, the pooled approach is better, but when the difference in the frontier increases, then the contemporaneous approach is the best.

In addition to the Simar and Wilson approach, Banker et al. [41] have shown that for certain cases, a simple DEA + OLS approach will outperform the Simar and Wilson approach. In our second stage analysis, we will therefore start with the DEA + OLS approach and compare the result with a bootstrapped truncated regression.

### Data

The hospital activity data used as output data have been provided by the Norwegian Patient Registry. As noted above, each output is a weighted sum of the total DRG-points in that category. To avoid confusing changes in activity with changes in the DRG-system and the associated cost weights, the activity data has been regrouped using the same grouping logic, using 2018 as the reference year. In addition to this, we use fixed cost-weights based on the average DRG reimbursement for 2019 (or the newest available data). This way of regrouping longitudinal hospital activity data with a common DRG- and price weight is a novel approach first introduced by Anthun et al. [15]. These measures enhance the comparability over time, and we avoid some of the potential errors that studies using longitudinal hospital activity data are prone to. Concerning the input data, operating costs are derived from The Norwegian Directorate of Health, while capital costs are derived from Statistics Norway.

The data for the second stage analysis are derived from the following sources: The variable for the budget result is obtained from official documents and is further calculated as a percentage of total operating costs. Since the health trusts have budgeted with both positive and negative results, and thus includes both positive and negative values, we have included it as a squared term. This is done to capture potential non-linear relationships. The competition variable is calculated based on the hospital activity data from the Norwegian Patient Registry. The personnel mix variable stems from Statistics Norway. Data for the hospital structure system variable stems from manual counting, and it is DRG-adjusted with data from the Norwegian Patient Registry. The data on research output is from The Nordic Institute for Studies in Innovation, Research and Education, and is available from the Norwegian Government’s website [42]. This is DRG-adjusted the same way as the hospital structure variable.

## Results

### Stage 1: Results from DEA and MI

**Fig. 1:**
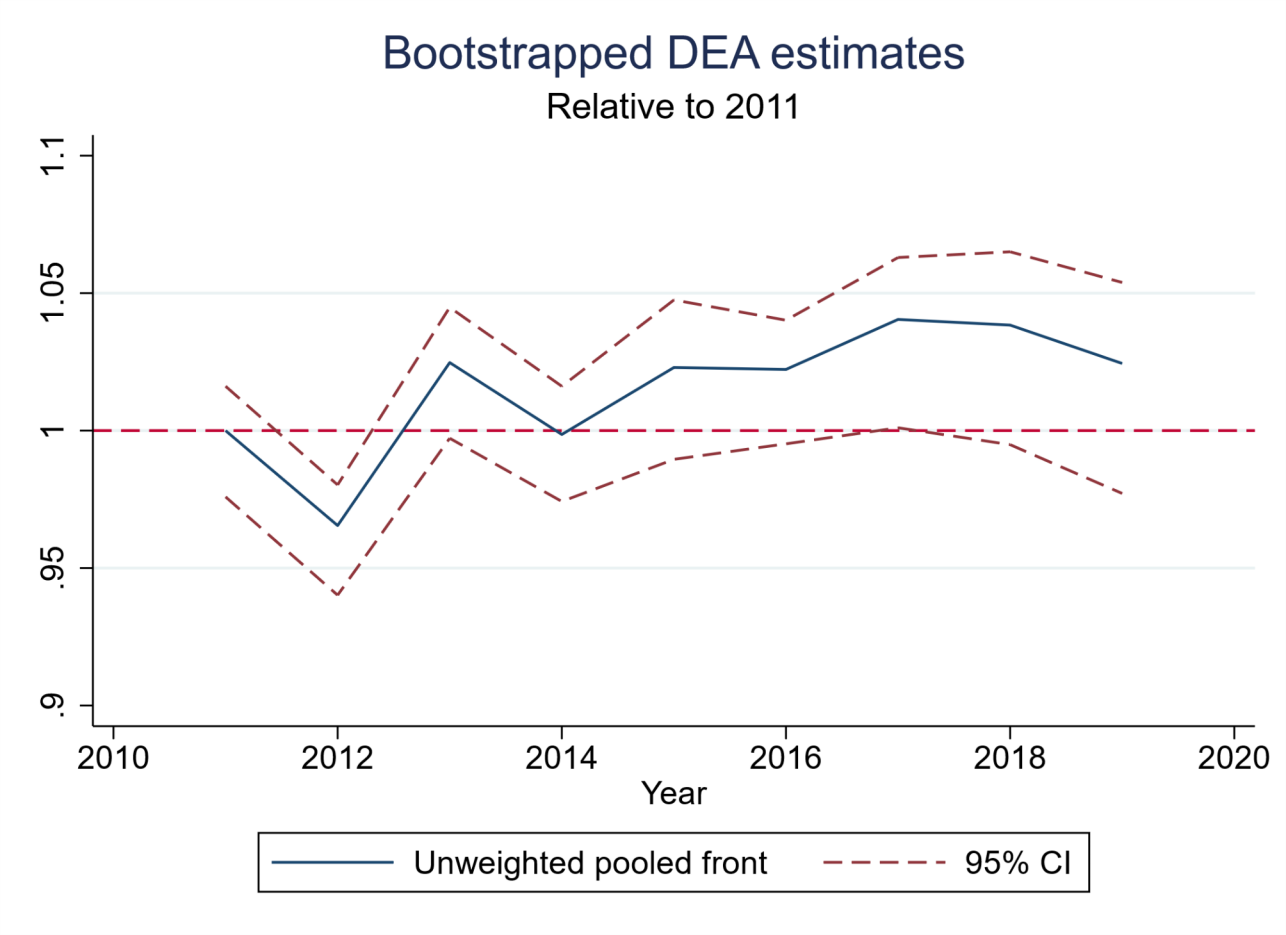
Bootstrapped DEA estimates with 95% confidence intervals. Numbers are relative to 2011

The result from the pooled DEA is presented in Fig. 1 below, and the MI is presented in Table 3. Both the DEA and the MI are run with bootstrapping as proposed by Simar and Wilson [43, 44]. We used 2000 bootstrapping iterations.

Using 2011 as a reference year, we see that there has been an overall increase in efficiency of approximately 2.5% from 2011 to 2019. The changes are, however, not within the 95% CI. This equals an average annual increase of approximately 0.27% points.

The results from the Malmquist productivity index are shown in Table 3. There was a total productivity growth in all the years, except from 2011 to 2012, and from 2013 to 2014. Four of these are significant (12/13, 14/15, 15/16, 16/17). In these years, the average hospital was moving closer to the frontier. Two of the years (11/12 and 13/14) had a significant score lower than 1.0, indicating that the average hospital was moving away from the frontier.

The score for the front shift was above 1.0 in five out of the eight year-shifts. However, in all of these five years, the confidence intervals were too wide to show any evidence of an actual frontier shift. We use this lack of evidence as an argument for basing the analysis in stage 2 on the pooled estimation of DEA, rather than an annual.

**Table 4:** Results from the Malmquist Productivity Index. Confidence intervals in parentheses

Year	M (productivity growth)	MC (catching up)	MF (front shift)
11/12	0.956 (0.947–0.959)	0.957 (0.853–1.024)	0.998 (0.925–1.097)
12/13	1.092 (1.090–1.095)	1.033 (0.976–1.119)	1.055 (0.965–1.111)
13/14	0.988 (0.976–0.992)	1.010 (0.912–1.063)	0.976 (0.923–1.065)
14/15	1.032 (1.009–1.044)	1.019 (0.956–1.081)	1.012 (0.945–1.071)
15/16	1.013 (1.008–1.020)	0.992 (0.906–1.050)	1.020 (0.957–1.101)
16/17	1.010 (1.001–1.020)	0.999 (0.914–1.062)	1.009 (0.942–1.093)
17/18	1.006 (0.998–1.014)	1.014 (0.924–1.067)	0.990 (0.934–1.076)
18/19	1.001 (0.994–1.007)	0.996 (0.919–1.031)	1.005 (0.968–1.076)

### Stage 2: Regression

The second stage of analysis is presented in Table 4. Model 1 is a simple DEA + OLS model as suggested by Banker et al. [41], while model 2 is inspired by Simar and Wilson [39] by using truncated regression with bootstrapping on bias-corrected DEA estimates. Both models are run with RHA- and time fixed-effects with clustering upon the health trust to avoid serial correlation. The results from the second stage regression analyses are presented in Table 5.

**Table 5:** Regression analyses

VARIABLES	Model 1	Model 2
	OLS regression	Truncated regression
Budgeted result	0.00541	0.00771
	(-0.00320–0.0140)	(-0.00799–0.0234)
Budgeted result squared	0.00242**	0.00358
	(0.000430–0.00440)	(-0.00106–0.00822)
Market concentration (HHI)	-0.191	-0.252
	(-0.664–0.282)	(-1.435–0.932)
Personnel mix	-0.00772**	-0.0105*
	(-0.0153 - -0.000159)	(-0.0229–0.00179)
Hospital structure (DRG adjusted)	0.106***	0.130**
	(0.0456–0.167)	(0.00724–0.252)
Research output (DRG adjusted)	0.00735**	0.00938
	(0.000210–0.0145)	(-0.0103–0.0291)
Constant	1.073***	1.146***
	(0.871–1.276)	(0.627–1.664)
Observations (health trust-years)	171	171
R-squared	0.582	0.579
RHA FE	YES	YES
Year FE	YES	YES

Robust confidence intervals in parentheses

*** *p* < 0.01, ** *p* < 0.05, * *p* < 0.1

**Fig. 2:**
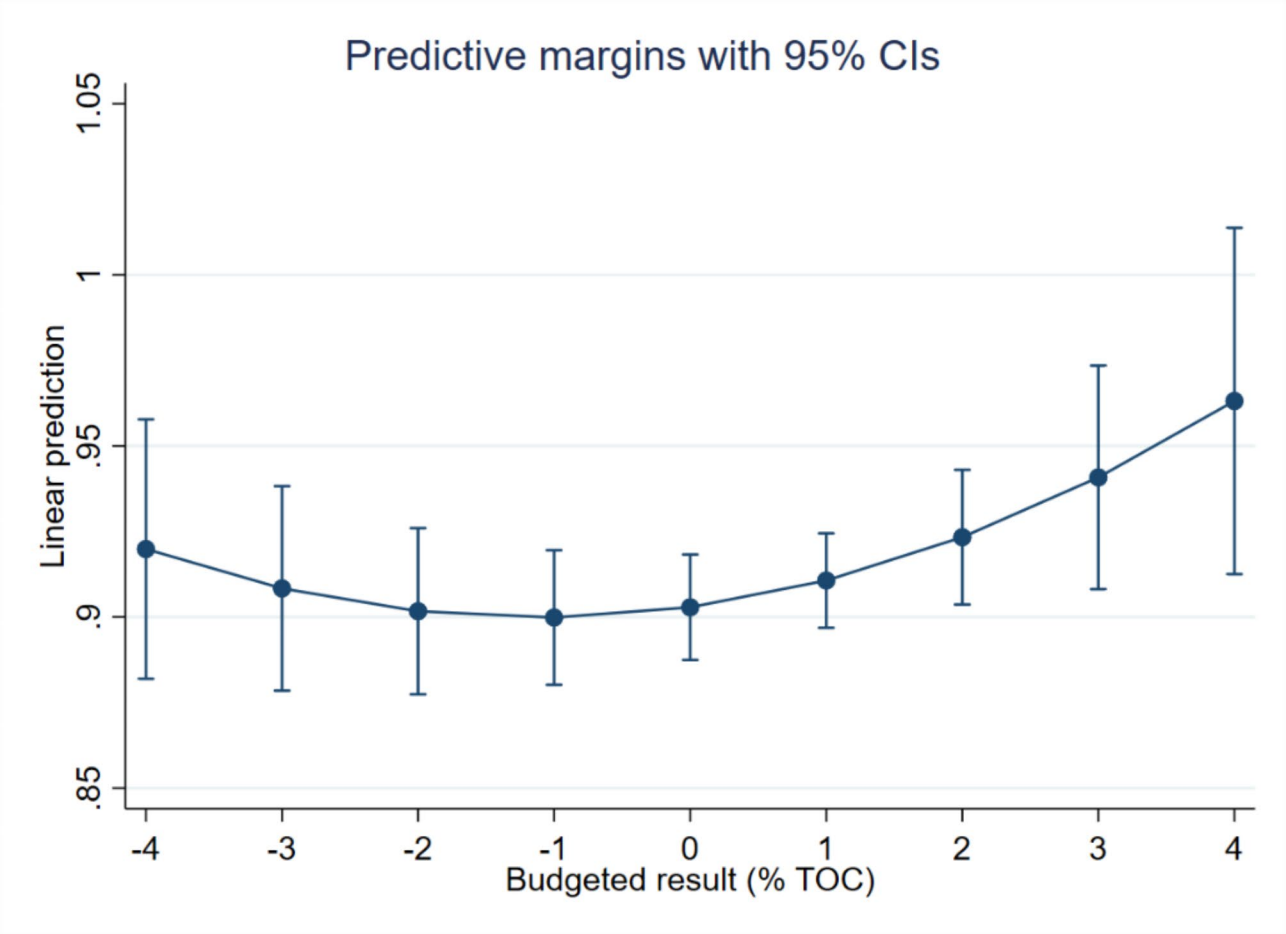
Predicted relationship between budgeted result and health trust efficiency

Firstly, we can see that all the coefficients are somewhat higher in model 2. The models also have a relatively high explained variance of approximately 58%. We begin by looking at the *budgeted result*. In model 1, the relationship between the squared variable and the dependent variable is statistically significant, thus indicating the presence of a non-linear relationship. We have graphed this relationship in Fig. 2. Here we see that budgeting with higher surpluses is associated with higher health trust efficiency, which is in line with what we expected. We also see that budgeting with a high negative result is also associated with an increase in efficiency. In model 2, we see that that the coefficients are of similar magnitude, but the confidence intervals are wider, making the relationship not significant and thus the finding from model 1 somewhat less robust.

Looking at the association between *market concentration* and efficiency, we see a negative association with increased competitive pressure. However, the wide confidence intervals of this variable indicate that this association is not statistically significant.

The *personnel mix* variable has a negative association with hospital efficiency, indicating that when the share of administrative personnel increases, the hospital efficiency is reduced. This association is statistically significant on the 0.05-level in model 1 and the 0.1-level in model 2. The *hospital structure* variable has a positive coefficient in both models, and this is statistically significant in both models. Thus, while there is an indication that an increase in the number of hospitals within a health trust is associated with increased efficiency. The DRG-adjusted *research output* which has a positive coefficient indicates a positive association between increased research output and efficiency. This is only statistically significant in model 1. Lastly, we also tested models controlling for the effect of both size and quality (measured as 30-day survival rate), but the inclusion of these variables did not lead to any substantial changes in the results. In the Supplementary Materials, we present a sensitivity analysis where we test for the inclusion of a lagged version of the budgeted result. This showed that the squared version of the budgeted result, has almost the same effect estimate as in the original models, but concerning the robustness of the models, the lagging of the variables removes a substantial share of the observations.

## Discussion

First, we see that hospital efficiency has increased over time. The annual change in efficiency is however not significantly higher that the efficiency score for 2011. The trend we see in this study is in line with the results in Anthun et al. [15] for four overlapping years. As most of the increase they observed occurred in the period immediately after the 2002 hospital ownership reform, it is perhaps not surprising that our finding of an annual growth of 0.27% points is substantially lower than Anthun et al.’s observed annual growth of 1.5% points. Furthermore, the results from the MI shows that there is productivity growth each year after 13/14, but the size of the growth is lower for each year. We find no clear evidence of neither catching up nor front shifts.

The 2002 reform forced the health trust to plan and finance their investments by running with a surplus, which was a clear use of incentivization to make the health trusts more efficient. We used the budgeted results to measure the health trust’s intentions to follow the incentives, with the assumption that when they budgeted for a larger surplus, they would also be more efficient in order to actually gain the surplus they budgeted for. The results from the second stage regression analysis in model 1 indicates that budgeting with a higher surplus (as a percentage of total operating costs) is associated with higher health trust efficiency, and thus confirms this assumption. What we also saw was that an increasingly larger *negative* budgeted result was associated with increased efficiency. A possible explanation of this is that when the health trusts see it necessary to budget with a negative result, despite the clear incentives to not do so, it leads to them having an increased discipline which increases the overall efficiency of the health trust. Since this association was only statistically significant in model 1, this relationship must be treated with some caution. However, we believe that this analysis has revealed some overall patterns that should be further investigated with in-depth case studies to reveal some of the potential mechanism that possibly links the budgeted results of the health trusts with their overall efficiency. In this study we are only looking at overall associations and not the causal relationships, and we can therefore not be sure of the potential underlying causal mechanism.

Our second main variable of interest was competition. We did not find any association between market concentration and efficiency. Based on the past research on the role of competition in the Norwegian hospital sector, we did not necessarily expect to find an association between competition and efficiency. However, it is still important to investigate how this variable is associated with efficiency, as the reforms in 2001 and 2015 had an intention of making the health trusts compete for patients as a means for increasing efficiency. Looking at the direction of the coefficient, however, we see that contrary to the theoretical assumptions of how organizations are expected to respond to competition, we see that increased competition is associated with lower levels of efficiency. Our findings here are consistent with German studies [23, 24].

There was a negative association between administrative staff and hospital efficiency. This can be interpreted such that less administrative staff opens up for more core medical staff, which in turn does the actual patient treatment. This finding is in line with the findings from England suggesting that the proportion of medical staff is positively associated with increased labor productivity [28]. However, it is not clear whether this is directly related to earlier findings from Norway showing that more administrative tasks are being done by physicians [45, 46]. A higher share of administrative staff could indicate a reduced need for the physicians to do administrative work, and thus more time for actual patient treatment, which in turn should lead to higher efficiency. That would on the other side lead to a lower share of the core personnel that does the patient treatment. Although some of the physicians’ administrative tasks possibly could have been done by the administrative staff, increased demands for documentation leads to a large body of administrative work that has to be done by the physicians themselves [45]. Since we do not have data on physicians’ working hours, we cannot draw any conclusions about the association between administrative staff and physicians’ administrative tasks.

The hospital structure variable has a positive effect in both models. This indicates that the more hospitals the health trust has per DRG point, are associated with increased efficiency. Large health trusts with few subunits will according to this analysis have lower efficiency than small. This effect also remained the same when controlling for the size of the hospital (not reported here), which indicates that this effect is not directly determined by the size of the health trust. The possibility for the health trusts, and the RHAs to change the number of hospitals has some structural limits including both geography and population.

This study provides some findings about the long-term effects of central policy tools on hospital efficiency. We have provided some novel approaches for operationalizing aspects of NPM. Even though there is great heterogeneity between different health systems, and how various NPM-reforms have been implemented in different countries, we still believe that this study still provides findings that are relevant for researchers in other countries as well. In future research, it would therefore be of interest to see the same set of variables used in a similar study in another country. This study has focused on some overall trends for the hospital sector as a whole but as mentioned earlier, it is clear that this requires further and more in-depth studies to gain more knowledge of the potential underlying mechanisms of how NPM-reforms are (or not are) associated with efficiency.

There are some limitations of the study that needs to be addressed. First, this study has no intentions of uncovering causal mechanisms concerning how NPM-features affect the hospital sector. We are merely looking for associations between the NPM-features and efficiency levels. Second, statistical analyses will in most cases be more robust with a larger amount of data to back up the results. Since the Norwegian hospital sector is small measured in the number of health trusts there is a huge limitation on how large datasets one can get. On the other sides, this study has some distinct strengths as well. First, this study uses a common DRG-grouper for all years. This ensures a higher degree of reliability when comparing hospital activity across time, where changes in documentation, procedures, and technology are likely to occur [15]. This also applies to the use of fixed cost-weights. Had we not employed fixed-cost weight, some of the actual efficiency changes could possibly not have been captured by the analysis, since this removes potential time trends related to cost changes rather than efficiency changes, and also absorb some of the technological changes that affects the frontier. Second, we use bootstrapping to estimate bias-corrected efficiency scores. By doing this, we get a measure of the uncertainty of the estimates. Third, by employing a panel data approach with time-fixed effects, we can control for unobserved changes that vary over time.

## Conclusion

This study has provided an explorative analysis of how the efficiency in Norwegian health trust has developed over time, and its association with certain NPM-features. Although not statistically significant, we find through a DEA analysis that the overall efficiency between 2011 and 2019 has increased by 2.5% points, with an average annual increase of 0.27% points. The study finds an association between several factors and efficiency, including both budgeted result, personnel mix, and hospital structure. For one of the most distinct NPM-features, competition, we find no evidence of association. The latter finding might, however, be due to methodological reasons concerning the measurement of competition. Internationally, there is an extensive field of research focusing on hospital efficiency. However, there is a need for more research on how NPM reforms and specific NPM components relate to this as this still remains a debate both within and outside the world of academia. First, more in-depth case studies of individual health trust should investigate how they have responded both to the incentivization and competition measures introduced, as well as other NPM components. Such studies can help us gain more knowledge of the mechanisms related to how NPM reforms components have affected different aspects of hospital performance. Second, both in Norway, as well as internationally, future research should focus on new and innovative ways of operationalizing NPM in terms of quantitative variables. This is important because if we have reliable quantitative measurements of NPM variables, it would be easier to conduct systematic research across different settings.

### Electronic supplementary material

Below is the link to the electronic supplementary material.

Supplementary Material 1

## Data Availability

The data that support the findings of this study are available from the corresponding author upon reasonable request.

## Abbreviations

ABF: Activity–based financing
DEA: Data envelopment analysis
DMU: Decision–making unit
DRG: Diagnosis related groups
FTE: Full time equivalent
HHI: Herfindahl–Hirschman index
MDC: Major diagnostic category
MI: Malmquist index
NHS: National health service
NPM: New Public Management
OECD: The Organization for Economic Cooperation and Development
OLS: Ordinary least squares
RHA: Regional health authorities
