# Supplementary material for: New Public Management and hospital efficiency: the case of Norwegian public hospital trusts
Source: BMC Health Serv Res. 2024 Jan 5;24:36. doi: 10.1186/s12913-023-10479-7 (PMC10770877; doi:10.1186/s12913-023-10479-7)
Supplement: Supplementary file 1 — Supplementary Material 1 [file 12913_2023_10479_MOESM1_ESM.docx]

|  | Model 1 | Model 2 |
| --- | --- | --- |
| VARIABLES | OLS regression | Truncated regression |
|  |  |  |
|  |  |  |
| Budgeted result (lagged) | 0.00305 | 0.00312 |
|  | (-0.00474 - 0.0108) | (-0.0128 - 0.0190) |
| Budgeted result squared (lagged) | 0.00221* | 0.00314 |
|  | (-0.000159 - 0.00457) | (-0.00298 - 0.00926) |
| Market concentration (HHI) | -0.292 | -0.382 |
|  | (-0.773 - 0.188) | (-1.454 - 0.690) |
| Personnel mix | -0.00790* | -0.0104* |
|  | (-0.0160 - 0.000245) | (-0.0227 - 0.00187) |
| Hospital structure (DRG adjusted) | 0.0976*** | 0.118* |
|  | (0.0349 - 0.160) | (-0.00388 - 0.239) |
| Research output (DRG adjusted) | 0.00728** | 0.00919 |
|  | (0.000489 - 0.0141) | (-0.00921 - 0.0276) |
|  |  |  |
| Constant | 1.096*** | 1.174*** |
|  | (0.897 - 1.296) | (0.707 - 1.642) |
|  |  |  |
| Observations (health trust-years) | 152 | 152 |
| R-squared | 0.587 | 0.585 |
| RHA FE | YES | YES |
| Year FE | YES | YES |

Robust confidence intervals in parentheses

*** p<0.01, ** p<0.05, * p<0.1
